# ACT001 Inhibits TLR4 Signaling by Targeting Co-Receptor MD2 and Attenuates Neuropathic Pain

**DOI:** 10.3389/fimmu.2022.873054

**Published:** 2022-06-09

**Authors:** Tianshu Zhang, Cong Lin, Siru Wu, Sha Jin, Xiaodong Li, Yinghua Peng, Xiaohui Wang

**Affiliations:** ^1^ Laboratory of Chemical Biology, Changchun Institute of Applied Chemistry, Chinese Academy of Sciences, Changchun, China; ^2^ School of Applied Chemistry and Engineering, University of Science and Technology of China, Hefei, China; ^3^ State Key Laboratory of Natural and Biomimetic Drugs, Peking University, Beijing, China; ^4^ State Key Laboratory of Polymer Physics and Chemistry, Changchun Institute of Applied Chemistry, Chinese Academy of Sciences, Changchun, China; ^5^ Beijing Changping Huayou Hospital, Beijing, China; ^6^ Institute of Special Animal and Plant Sciences, Chinese Academy of Agricultural Sciences, Changchun, China; ^7^ Beijing National Laboratory for Molecular Sciences, Beijing, China

**Keywords:** neuropathic pain, toll-like receptor 4, myeloid differentiation protein 2, chronic constriction injury, ACT001

## Abstract

Neuropathic pain is a common and challenging neurological disease, which renders an unmet need for safe and effective new therapies. Toll-like receptor 4 (TLR4) expressed on immune cells in the central nervous system arises as a novel target for treating neuropathic pain. In this study, ACT001, an orphan drug currently in clinical trials for the treatment of glioblastoma, was identified as a TLR4 antagonist. *In vitro* quenching titrations of intrinsic protein fluorescence and saturation transfer difference (STD)-NMR showed the direct binding of ACT001 to TLR4 co-receptor MD2. Cellular thermal shift assay (CETSA) showed that ACT001 binding affected the MD2 stability, which implies that MD2 is the endogenous target of ACT001. *In silico* simulations showed that ACT001 binding decreased the percentage of hydrophobic area in the buried solvent-accessible surface areas (SASA) of MD2 and rendered most regions of MD2 to be more flexible, which is consistent with experimental data that ACT001 binding decreased MD2 stability. In keeping with targeting MD2, ACT001 was found to restrain the formation of TLR4/MD2/MyD88 complex and the activation of TLR4 signaling axes of NF-κB and MAPKs, therefore blocking LPS-induced TLR4 signaling downstream pro-inflammatory factors NO, IL-6, TNF-α, and IL-1β. Furthermore, systemic administration of ACT001 attenuated allodynia induced by peripheral nerve injury and activation of microglia and astrocyte *in vivo*. Given the well-established role of neuroinflammation in neuropathic pain, these data imply that ACT001 could be a potential drug candidate for the treatment of chronic neuropathic pain.

## 1 Introduction

Neuropathic pain is a chronic and pathological disease resulting from nerve injury or inflammation which remains poorly managed by currently available therapeutics (1). Most of these therapeutics target neurons (2). Recently considerable investigations demonstrate that glia also plays a key role in neuropathic pain outcomes (3, 4). Once the peripheral nerve is injured, glial cells become activated and release pro-inflammatory cytokines, chemokines, and other inflammatory mediators such as nitric oxide (NO), which contributes to the maintenance of neuronal central sensitization (4, 5). Therefore, resetting the activated glia to the resting state and blocking the neuroinflammation would be a useful intervention strategy for treating neuropathic pain.

Toll-like receptor 4 (TLR4) is a pattern recognition receptor (PRR), which is responsible for the recognition of pathogen-associated molecular patterns (PAMPs), damage-associated molecular patterns (DAMPs), and xenobiotic-associated molecular patterns (XMAPs) (6). Lipopolysaccharide (LPS), a component of the outer membrane of Gram-negative bacteria, is a natural ligand of TLR4 (6). In the central nervous system(CNS), TLR4 is primarily expressed on microglia (7), functioning mainly in the regulation of pro-inflammatory factors production. Injured sensory neurons may release extracellular matrix molecules and DAMPs, which are detected by TLR4 thus activating immunocompetent cells and exerting the influence on the neural pain (7, 8). Therefore, TLR4 antagonists could be potential therapeutics for treating neuropathic pain. However, there are rare TLR4 antagonist that cross the blood-brain barrier (BBB).

ACT001 (also known as dimethylaminomicheliolide, DMAMCL), derived from parthenolide (9), displays anti-tumor activities in various cancers, including hepatocellular carcinoma, breast cancer, and glioblastoma (10–13). As a promising drug for the treatment of glioblastoma, ACT001 has an excellent effect on restraining the growth of glioblastoma in Phase I clinical trials and now it is currently undergoing Phase II clinical trials (14, 15). ACT001, which can penetrate the blood–brain barrier (BBB) and accumulate in the brain, alleviates glial activation and neuroinflammation (16, 17). As a foreign substance in CNS, it is not surprising that ACT001 would perturb the CNS immunity (16, 17). TLR4 is the key PRR of innate immune system, which detects PAMPs (18), DAMPs (19) and XMAPs (20, 21). It would be interesting to explore whether ACT001 acts as a XAMP and can be sensed by myeloid differentiation 2 (MD2), an accessory protein of TLR4 responsible for the recognition of ligand. This study found that ACT001 bound to MD2 and inhibited LPS-induced formation of TLR4/MD2/MyD88 complex and the activation NF-κB and MAPKs, therefore suppressing LPS-induced pro-inflammatory factors. Moreover, intravenous injection of ACT001 attenuated allodynia induced by peripheral nerve injury and lumbar spinal cord dorsal horn expression of Iba-1 (a microglial activation marker) and GFAP (an astrocyte activation marker) *in vivo*. These data implicate that ACT001 has the potential for treating neuropathic pain.

## 2 Manuscript Formatting

### 2.1 Materials and Methods

#### 2.1.1 Materials

ACT001 was kindly provided by Tianjin Shangde Pharmaceutical Margin Technology Co., Ltd. Microglial BV-2 cells were obtained from China Center for Type Culture Collection. Ultrapure lipopolysaccharide (LPS), HEK Blue TLR4 293 cells, and HEK-Blue Selection were obtained from Invivogen. Phospha-Light™ SEAP Reporter Gene Assay System was purchased from Applied Biosystems. Dual-Glo Luciferase Assay System was purchased from Promega. Crystal violet, 2,3-diaminonaphthalene, protease inhibitor cocktails, and anti-β-actin antibodies were purchased from Sigma-Aldrich. Dulbecco’s Modified Eagle Medium (DMEM), TRIzol, RIPA buffer were purchased from Thermo Fisher Scientific. Fetal bovine serum was purchased from PAN-Seratech. RNeasy Mini Kit, RT² Easy First Strand cDNA Synthesis Kit, PCR primers and SYBR Green PCR Master Mix were obtained from Qiagen. Primary MD2 antibody, anti-Iba1 antibody, anti-TLR4 antibody and anti-GFAP antibody were purchased from Abcam. Primary antibodies targeting MyD88, p38 MAPK, NF-κB p65, ERK (1/2), IKK-β, SAPK/JNK, phospho-NF-κB p65, phospho-ERK(1/2), phospho-SAPK/JNK, phospho-IKK-α/β, and phospho-p38 MAPK antibodies were obtained from Cell Signaling Technology.

#### 2.1.2 Fluorescence Titrations of MD2 With ACT001

MD2 expression and purification were performed as described previously (22, 23). Fluorescence titrations of MD2 with ACT001 were performed on a Cary Eclipse spectrofluorometer. All measurements were carried out at room temperature using a 2×10 mm quartz cell with MD2. The fluorescence titration was carried out at a wavelength of 280 nm to excite the Tyr and Trp residues in MD2. Emission at 310-450 nm was measured. 0.5 μM MD2 was titrated with different concentrations of ACT001 and fluorescence intensity at 337 nm was plotted against ACT001 concentration.

#### 2.1.3 Saturation Transfer Difference (STD) NMR Measurement

MD2 was prepared in a phosphate buffer in D_2_O (75 mM potassium phosphate, 150 mM sodium chloride, pH 7.5)and ACT001 was dissolved in DMSO-d6 (50 mM) as stock solution. Saturation transfer difference NMR spectroscopy experiments were performed to investigate ligand-protein interactions. NMR spectra were acquired at 25 °C in a Bruker Avance III-600 MHz (proton frequency) spectrometer with a conventional inverse 5 mm probe head with z-gradients using standard Bruker pulse programs. Samples containing 400 μM ACT001 in the absence or presence of MD2 (4 μM) in D_2_O buffer were used for NMR spectra data acquisition.

#### 2.1.4 Cellular Thermal Shift Assay (CETSA)

Cellular thermal shift assay (CETSA) was performed as described (21).

#### 2.1.5 *In Silico* Simulations

##### 2.1.5.1 System Preparation and Docking

The structure of ACT001 was drawn through Gauss View 6 (24) and optimized by Gaussian 09 (25) software using the B3LYP density functional method and 6-31G (d,p) basis set. The X-ray structure of MD2 was extracted from TLR4/MD2 complex (PDB ID: 2Z64) and was used for molecular docking and molecular dynamics simulations. Missing hydrogen atoms of MD2 were added by Maestro under pH 7.0 (26). Molecular docking was conducted through AutodockVina 1.1.2 in a box of 50 × 60 × 50 Å^3^, which covers MD2 protein completely (27). The most favorable binding site was searched and located by the Iterated Local Search Globule Optimizer (28, 29). MD2 was considered rigid and ACT001 was regarded as semi-flexible during molecular docking. Ten docking poses were generated by AutodockVina 1.1.2 and ranked according to their affinity with MD2. Of all the docking poses, the pose with the best affinity to MD2 was chosen for further simulations.

##### 2.1.5.2 Molecular Dynamics Simulation

MD2 alone (apo-MD2) and the best docking pose of MD2 interacting with ACT001 were further studied through molecular dynamics simulations by the NAMD2.12 package (30) with AMBER ff03 force field (31, 32). R.E.D was used to optimize and fit the atomic charges of ACT001 based on the quantum mechanics calculations (33). The general AMBER force field (GAFF) was used to treat other atomic parameters (32). A TIP3P model of a water box was used to solvate all solutes with a distance of 10 Å between the protein and the edge of the box. Na^+^ and Cl^-^ ions were added to neutralize the system with a concentration of 0.15 M. Energy minimization was performed for 5000 steps first and the system was heated to 310 K in 310 ps with 1 ns equilibration. The system was further run in the isothermal-isobaric (NPT) ensemble at a temperature of 310 K for 400 ns. SHAKE algorithm was used to restrain all bonds involving hydrogen (29). Calculations of long-range electrostatic interactions were performed by the Particle-mesh Ewald (PME) summation method (34). Langevin dynamics was used to keep the temperature of the system at 310 K with the collision frequency of 5 ps^–1^ and the pressure was set at 1 atm with Nosé–Hoover Langevin piston method (35).

The RMSD (root-mean-square deviation) and RMSF (root-mean-square fluctuation) analyses were performed through VMD (36) and Bio3D package (37), respectively. The interactions between MD2 and ACT001 were analyzed by LigPlot^+^ (38) and PyMol (39) software. The ratio of hydrophobic SASA (solvent accessible surface areas) in buried SASA was calculated as specified before (20).

#### 2.1.6 Nitric Oxide (NO) Assay

BV-2 cells were cultured in supplemented DMEM (10% FBS, 50 U/mL penicillin, and 50 μg/mL streptomycin) and seeded at a density of 4×10^4^ cells per well in 96-well plates. After overnight incubation, media was aspirated and changed to DMEM media without FBS. Cells were then treated with LPS (200 ng/mL) and the indicated concentrations of ACT001. The NO concentration in the culture supernatant was determined by the 2,3-diaminonaphthalene-based fluorescent method as described (23).

#### 2.1.7 BV-2 Cells Morphology

BV-2 cells were cultured as above and treated with LPS (200 ng/mL) and 100 μM ACT001 for 6 h. Cell morphology images were collected by a Nikon microscope.

#### 2.1.8 Cell Viability Assay

Cellular viability was determined by the crystal violet staining method and CCK-8 Kit as described (23, 40).

#### 2.1.9 Secreted Embryonic Alkaline Phosphatase (SEAP) Assay

SEAP assay was performed as described (40).

#### 2.1.10 Dual-Luciferase NF-κB Reporter Assay

Dual-luciferase NF-κB reporter assay was performed as described (40).

#### 2.1.11 Co-Immunoprecipitation (Co-IP)

BV-2 cells were seeded at 4×10^6^ cells/dish in 100 mm culture dishes. After 24 h incubation, cells were stimulated by LPS (200 ng/mL) and indicated concentrations of ACT001 for 1 h. Cells were washed twice with ice-cold PBS and lysed in 1 mL Co-IP lysis buffer (25 mM Tris pH 8.0, 150 mM KCl, 5 mM EDTA, 0.5% NP-40) with a complete protease inhibitor cocktail, 1 mM DTT, and 1 mM PMSF by incubating on ice for 30 min. Cell supernatant was collected *via* centrifugation at 12,000 g at 4 °C for 12 min and incubated with corresponding primary antibody at 4 °C overnight. Washed magnetic beads were then incubated with the samples at room temperature for 1 h. The magnetic beads were washed twice with PBS and boiled with 50 μL 2 × SDS sample buffer at 100 °C for 8 min for immunoblotting.

#### 2.1.12 Immunoblotting

Immunoblotting was performed as described (23).

#### 2.1.13 qRT-PCR

BV-2 cells were seeded at a density of 4×10^5^ cells/well in 6-well plates. After overnight incubation, BV-2 cells were treated with LPS (200 ng/mL) and indicated concentrations of ACT001 for 6 h. Total RNA was isolated from BV-2 cells using TRIzol reagent and cDNA was generated with an oligo (dT) primer. Primer sequences are shown in **Table 1**. The ribosomal protein L27 gene RPL27 was used as the internal control. qPCR was performed on a TOptical Real-Time qPCR Thermal Cycler (Analytik Jena, Thuringia, Germany) using the SYBR Green method. The data were analyzed by the 2^−ΔΔCT^ method and were normalized to RPL27.

**Table 1:** Primer sequences of iNOS, IL-1β, TNF-α, IL-6 and RPL27.

Gene		Sequence (5’-3’)
iNOS	Forward	GGGCTGTCACGGAGATCAATG
	Reverse	GCCCGGTACTCATTCTGCATG
IL-1β	Forward	CCACCTTTTGACAGTGATGA
	Reverse	GAGATTTGAAGCTGGATGCT
IL-6	Forward	TAGTCCTTCCTACCCCAATTTCC
	Reverse	TTGGTCCTTAGCCACTCCTTC
TNF-α	Forward	CCCTCCAGAAAAGACACCATG
	Reverse	GCCACAAGCAGGAATGAGAAG
RPL27	Forward	AAGCCGTCATCGTGAAGAACA
	Reverse	CTTGATCTTGGATCGCTTGGC

#### 2.1.14 *In Vivo* Study

##### 2.1.14.1 Animals and Drug Treatment

Pathogen-free adult male Sprague-Dawley rats (300-350 g) were used in all experiments (Liaoning Changsheng Biotechnology, China). Rats were housed in temperature-controlled (20 ± 2 °C) and light-controlled (12-h light-dark cycle; lights on at 7:00 am) rooms with standard rodent food and water available ad libitum and allowed to habituate to the holding facility for ≥1 week before experimentation. All the animal-handling procedures were approved by the Institutional Animal Care and Use Committee (IACUC) of Changchun Institute of Applied Chemistry, Chinese Academy of Sciences (CIAC2021-0026).

Animals were randomly divided into three groups. Rats in sham group (n = 6) and CCI group (n = 9) were intravenously administrated with 0.9% saline, while rats in the CCI + ACT001 group (n = 9) were intravenously administrated with 50 mg/kg ACT001 (dissolved in 0.9% saline), once a day from the 2^nd^ day to 42^nd^ day after surgery.

##### 2.1.14.2 CCI Induced Neuropathic Pain

Neuropathic pain was induced using chronic constriction injury (CCI) surgery as described previously (41). Briefly, rats were anesthetized and maintained with isoflurane. The left sciatic nerve was gently exposed. Four ligations were tied loosely around the sciatic nerve with sterile chromic gut sutures. The sham group animals were treated with the same surgery but without the ligation. All animals were monitored postoperatively until fully ambulatory before returning to their home cage and checked daily for any sign of infection. No such cases occurred in this study.

##### 2.1.14.3 Mechanical Allodynia

Animals received at least two days of habituation in the test environment before baseline testing. The nociceptive behavior was monitored 1 day before surgery and 10, 14, 17, 21, 24, 28, 31, 35, and 42 days after surgery. Rats’ weight was collected every two weeks to monitor animal health state. The stimulus with Von Frey filaments, ranging from 0.6 to 26 g, was applied to the plantar surface of the hind paw. The paw withdrawal threshold was accessed *via* the up-down method using the Chaplan formula (42).

##### 2.1.14.4 Immunofluorescence

Following the final behavioral testing, rats were anesthetized and perfused through the ascending aorta first with isotonic saline and then with fresh 4% paraformaldehyde in 0.1 M phosphate buffer (pH 7.4). The rat was decapitated, and the lumbar spinal cords (L4-L6) were removed immediately, immersed continuously in the 4% paraformaldehyde at 4 °C overnight. The spinal cord tissue was dehydrated with ethanol gradient, embedded in paraffin, and then sliced at a thickness of 4 μm. Paraffin-processed tissues were deparaffinized in xylene and rehydrated with a graded alcohol solution. The sections were placed in 0.01 M citrate buffer (pH 6.0) and heated in a microwave oven for hot repair antigen (43, 44). These sections were incubated with goat serum at 37 °C for 20 min and then with a mixture of rabbit-anti-Iba-1 monoclonal antibody and mouse-anti-GFAP monoclonal antibody at 4 °C overnight. After three washes with PBS, the sections were incubated with secondary antibody conjugated with Alexa-488 or 647 for 1 h in the dark. Ultimately, followed by three washes with PBS twice for 5 min each, the sections were counterstained with DAPI and examined under a confocal microscope.

#### 2.1.15 Statistical Analysis

Origin 8 was used for the plotting of the data and statistical analysis. Non-linear Logistic regression was used to plot and analyze concentration-response curves and to obtain IC_50_. For the analyses of qRT-PCR data, immunoblotting data, von Frey test and quantification of immunofluorescence, an unpaired Student t-test was used for comparisons between two groups. Data are presented as mean ± SEM. P-value summary is mentioned on the bar of each figure. # P< 0.05; ## P< 0.01; ### P< 0.001 *versus* the control/sham group; *P < 0.05; **P < 0.01; ***P < 0.001 *versus* the LPS/CCI group. ns, not significant. P< 0.05 was considered statistically significant in all analyses.

### 2.2 Results

#### 2.2.1 Biophysical Binding of ACT001 With MD2

**Figure 1:**
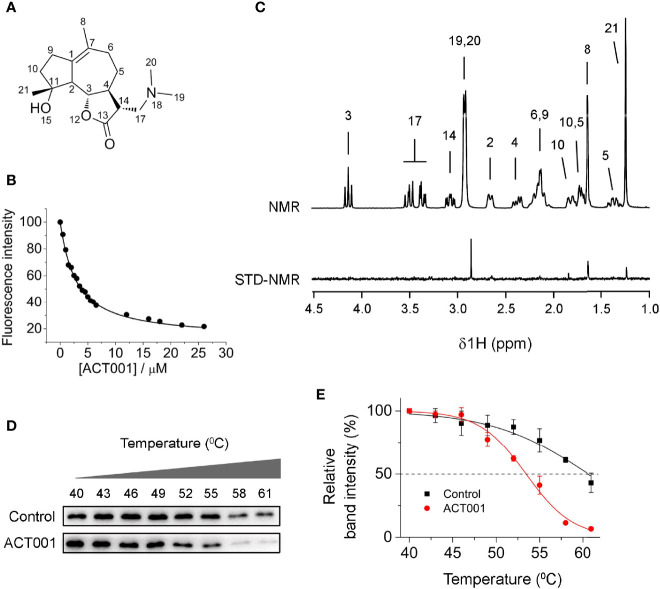
ACT001 binds to MD2. **(A)** The chemical structure of ACT001. **(B)** Titration curve of MD2 intrinsic fluorescence with the increasing ACT001. 280 nm was chosen as the excitation and emission at 337 nm (peak position) was plotted against the titrated ACT001 concentration. A value of K_d_ = 2.8 ± 0.3 μM was derived by nonlinear least-square fit a one-site-binding model for the MD2-ACT001 interaction. **(C)** The upper panel corresponds to the NMR assignments of ACT001; the lower panel is the saturation transfer difference spectrum recorded for 400 μM ACT001 in the presence of MD2 (4 μM). **(D)** Cellular thermal shift assay of MD2 with ACT001. **(E)** Quantification of MD2 shown in panel **(D)** was made using immunoblotting. Three independent cell culture preparations were performed. All data are given as the mean ± SEM.

Besides its anticancer action, ACT001 (**Figure 1A**) has also been reported to alleviate neuroinflammatory responses in the CNS (16). However, the molecular target responsible for the immunosuppressive effects of ACT001 is not known. Herein the acting of ACT001 is hypothesized to be, at least in part, mediated by TLR4, which plays a fundamental role in regulating innate immunity. MD2, a co-receptor of TLR4, is responsible for ligand recognition (45). Fluorescence quenching titration of MD2 was first performed to explore the possible interaction of ACT001 with MD2 as the potential target for the inhibition of innate immune signaling. ACT001 caused the quenching of MD2 intrinsic fluorescence (**Figure 1B**). A dissociation constant K_d_ of 2.8 ± 0.3 μM was derived by the nonlinear least-square fitting of the titration curve of MD2-ACT001 interaction. Moreover, saturation transfer difference (STD) nuclear magnetic resonance (NMR) was employed to characterize transient receptor-ligand interaction. Only ligand protons that are in close contact with the receptor-binding site and receive magnetization transfer will appear in the difference spectrum (46, 47). As the difference spectra shown in **Figure 1C**, hydrogens of methyl at positions 8, 19, 20 and 21 exhibited the most favorable binding characteristics, which confirm the direct interaction between ACT001 and MD2. To explore whether MD2 is the endogenous target of ACT001, cellular thermal shift assay (CETSA) was performed. CETSA is based on the principle that drug binding leads to the thermal stability change of the target protein as reflected by the shift of its melting temperature (T_m_). ACT001 binding decreased the T_m_ value of MD2 by 6.4 ± 0.9 °C (**Figures 1D, E** **)**, which indicates ACT001 directly binds to MD2 in the cellular context. Taken together, these biophysical binding characterizations show MD2 is a direct target of ACT001.

#### 2.2.2 Computational Simulations of ACT001 Binding to MD2

**Figure 2:**
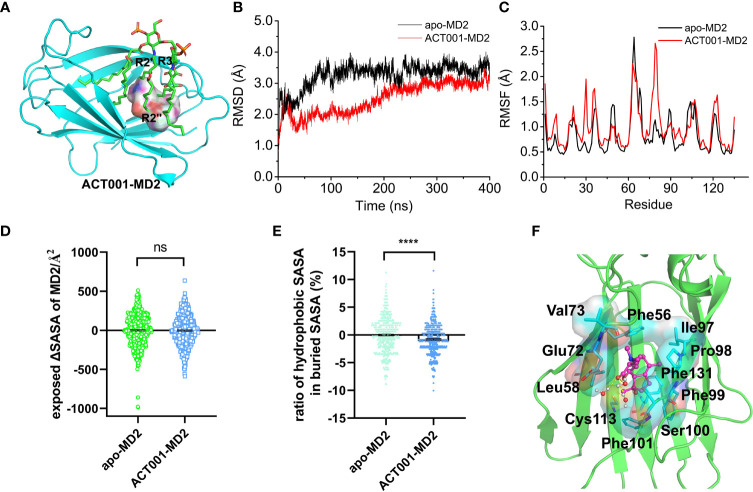
*In silico* simulation of ACT001 interacting with MD2. **(A)** Overlap of the best docking pose of ACT001 and lipid A in MD2. ACT001 occupied the LPS binding location (acyl chains R3, R2’ and R2’’). Lipid A was extracted from the active state of TLR4/MD2/LPS complex (PDB ID: 3VQ2) after aligning with ACT001-docked MD2. MD2 was shown as a cyan cartoon, lipid A as green sticks, and ACT001 as surface. **(B)** Time evolution of the RMSD of MD2 (apo-MD2) and ACT001 bound MD2 (ACT001-MD2) during the MD simulations at 310 K. **(C)** Time evolution of RMSF of MD2 and ACT001 bound MD2 during the MD simulations at 310 K. **(D)** The changes of the exposed SASA of MD2 upon binding with ACT001. Data were calculated based on the last 20 ns equilibrated MD trajectories at 310 K. ns, not statistically significant. **(E)** The ratio of the hydrophobic SASA in the buried SASA of MD2. Data were calculated based on the last 100 ns equilibrated MD trajectories at 310 K ****P< 0.0001. **(F)** The representative binding mode of ACT001 with MD2 at 310 K after molecular dynamics simulation.ACT001 was shown as a balls-stick model. MD2 was shown as a cartoon model. Key residues of MD2 in interacting with ligands were shown as stick and surface models labeled with residue names. Hydrogen bonds were shown as dashed lines in yellow. Water molecule interacting with ACT001 was represented as a balls-stick model.

In order to investigate how ACT001 interacts with MD2, molecular docking and molecular dynamics simulation were conducted. ACT001 was found to dock into the conserved hydrophobic cavity and overlap with the space of R2’, R3 and R2’’ chains of Lipid A in MD2, therefore hindering the binding of LPS to MD2 (**Figure 2A**). The best docking pose was refined using molecular dynamics simulations. The root-mean-square deviation (RMSD) analysis of backbone atoms of apo-MD2 and MD2 bound with ACT001 showed that both systems reached stable states during 400 ns simulations (**Figure 2B**). To investigate the flexibility changes caused by ACT001, the root-mean-square fluctuation (RMSF) analysis was conducted with the last 100 ns equilibrated trajectories. The binding of ACT001 rendered most regions of MD2 to be more flexible (**Figure 2C**), indicating that ACT001 destabilizes MD2. This result is consistent with the experimental CETSA data. The exposed solvent-accessible surface areas (SASA) of MD2 (**Figure 2D**) did not change upon interacting with ACT001. Interestingly, further analysis showed that ACT001 binding decreased the percentage of hydrophobic area in the buried SASA of MD2 (**Figure 2E**). It should be noted that the hydrophobic residues prefer to be buried inside owing to the hydrophobic interactions to stabilize the apo-MD2 (48). These *in silico* simulation results explicitly explain that ACT001 binding decreases MD2 stability.

The detailed binding mode of ACT001 with MD2 was subsequently investigated. **Figure 2F** shows the representative pose of ACT001 binding to MD2 after the molecular dynamic equilibration. ACT001 formed hydrogen bonds with surrounding water molecules and formed hydrophobic interactions with residues of MD2. Specifically, methyl at position 8 of ACT001 interacted with Ile97 and Pro98; methyl groups at positions 19 and 20 of ACT001 formed interactions with Val73; methyl at position 21 of ACT001 interacted with Phe101 and Cys113. These are consistent with STD-NMR results. In addition, Phe56, Leu58, Glu72, Phe99 and Phe131 were also found to form hydrophobic interactions with ACT001. All these *in silico* simulation results confirmed that ACT001 interacted with MD2 and decreased the stability of MD2.

#### 2.2.3 ACT001 Inhibits TLR4 Signaling and LPS-Induced Pro-Inflammatory Factors

**Figure 3:**
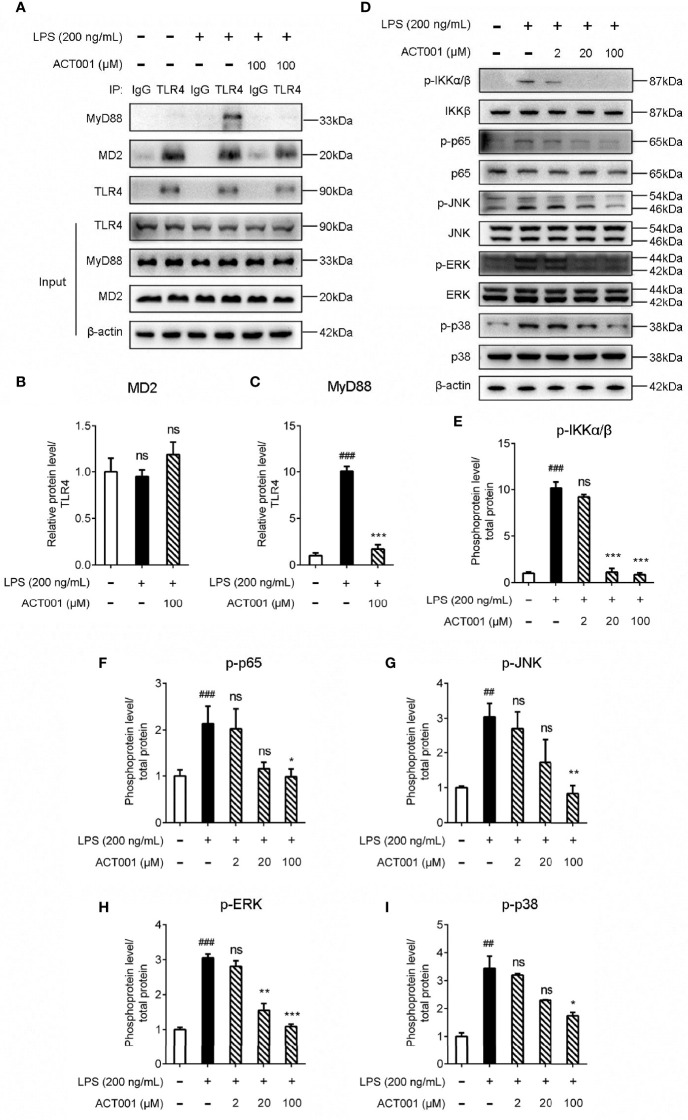
Cellular characterizations of ACT001 on TLR4 signaling. BV-2 cells were administered with 200 ng/mL LPS and the indicated concentration of ACT001 for 1 h. **(A-C)** Co-immunoprecipitation of anti-TLR4 antibody, MD2, TLR4, and MyD88 were detected by immunoblotting. **(D-I)** The effect of ACT001 on LPS-induced phosphorylation of IKKβ, p65 and MAPKs. The total protein level of IKKβ, p65 and MAPKs was set as reference. All experiments were performed three times independently, and data were given as the mean ± SEM. ^##^P< 0.01, ^###^P< 0.001 *versus* the control; *P< 0.05, **P< 0.01, ***P< 0.001 *versus* the LPS group; ns, not significant.

TLR4 activation leads to the recruitment of myeloid differentiation primary response protein 88 (MyD88) to activate NF-κB and MAPKs. Immunoprecipitation and immunoblotting were employed to measure the effect of ACT001 on TLR4 downstream signaling. As shown in **Figures 3A-C**, ACT001 inhibited LPS-induced MyD88 recruitment of TLR4 and significantly suppressed the formation of TLR4/MD2/MyD88 complex. LPS induced the phosphorylation of IKKβ, p65, JNK, ERK as well as p38 and ACT001 significantly inhibited LPS induced phosphorylation of these TLR4 signaling factors in a concentration-dependent manner (**Figures 3D-I**).

**Figure 4:**
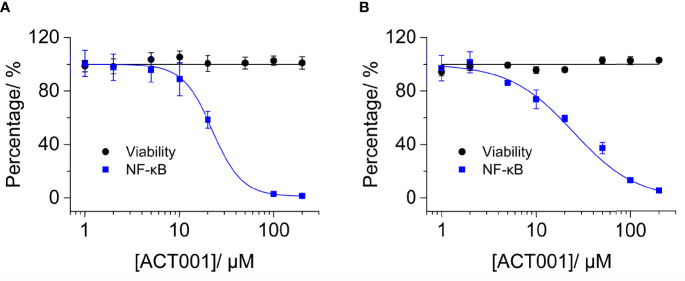
ACT001 inhibits LPS-induced NF-κB activation. **(A)** HEK Blue TLR4 293 cells, which over-express human CD14, TLR4, and MD2, were stimulated with 20 ng/mL LPS and indicated concentrations of ACT001. The NF-κB activity was determined by SEAP assay and cellular viability was measured by CCK-8 Kit. **(B)** BV-2 NF-κB luciferase reporter cells were treated with 200 ng/mL LPS and the indicated concentrations of ACT001. After 24 h of incubation, the NF-κB activity was determined by the Dual-Glo luciferase assay and cellular viability was measured by crystal violet staining. All experiments were performed 3 times independently and data were given as the mean ± SEM.

To further quantitatively investigate the effect of ACT001 on TLR4 signaling NF-κB activity, HEK TLR4 cell line with a SEAP reporter gene, under the control of NF-κB responsive element, was used. ACT001 was found to inhibit LPS-induced NF-κB activation in a dose-dependent manner, with an IC_50_ of 22.4 ± 0.3 μM while no apparent cellular toxicity of ACT001 was observed within 200 μM (**Figure 4A**). In addition to HEK based NF-κB reporter cell, the effect of ACT001 on NF-κB activity in BV-2 cells, which reproduces many of the responses of immunocompetent primary microglia with high fidelity (49),was also examined. ACT001 inhibited LPS-induced NF-κB activation in BV-2 cells in a dose-dependent manner with an IC_50_ of 24.1 ± 1.3 μM without apparent cellular toxicity within 200 μM (**Figure 4B**). These data clearly show that ACT001 inhibits TLR4 signaling NF-κB activation.

**Figure 5:**
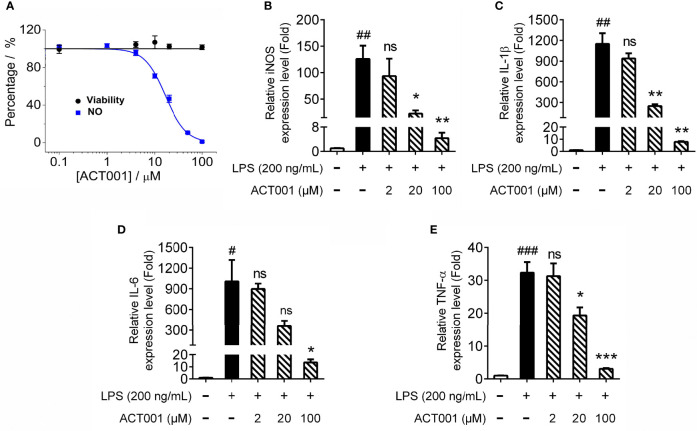
ACT001 inhibits LPS-induced pro-inflammatory factors overproduction. **(A)** BV-2 cells were administered with LPS and indicated concentrations of ACT001. After 24 h incubation, the effects of ACT001 on LPS-induced NO and cellular viability were measured. **(B-E)** BV-2 cells were administered with 200 ng/mL LPS and indicated concentrations of ACT001. After 6 h incubation, the effects of ACT001 on LPS induced iNOS **(B)**, IL-1β **(C)**, IL-6 **(D)** and TNF-α **(E)** mRNA were measured. All the data represented the mean ± SEM; n, number of independent cell culture preparations = 3. ^#^P< 0.05, ^##^P< 0.01, ^###^P< 0.001 versus the control; *P< 0.05,**P< 0.01, ***P< 0.001 *versus* the LPS group; ns, not significant.

The pro-inflammatory mediators are downstream effectors of TLR4 innate immune responses. ACT001 inhibited LPS induced nitric oxide (NO) overproduction in BV-2 cells in a concentration-dependent manner with an IC_50_ of 16.0 ± 1.2 μM (**Figure 5A**). No apparent cellular toxicity of ACT001 was observed, even at the concentration of 100 μM, which eliminates the possibility of the observed inhibition of TLR4 signaling by ACT001 was owing to cell death (**Figure 5A**). qRT-PCR was performed to measure the effect of ACT001 on LPS-induced pro-inflammatory factors iNOS, IL-1β, IL-6 and TNF-α mRNAs expression. ACT001 suppressed LPS-induced iNOS (**Figure 5B**), IL-1β (**Figure 5C**), IL-6 (**Figure 5D**) and TNF-α (**Figure 5E**) mRNA expression in a concentration-dependent manner. The LPS-induced cell body was enlarged, and the percentage of amoeboid-like microglia increased significantly, while ACT001 markedly reversed the LPS induced morphological changes in BV-2 cells (**Supplementary Figure 1**). Together, these cellular signaling characterizations demonstrate that ACT001 inhibits the formation of TLR4/MD2/MyD88 complex and LPS-induced activation of NF-κB and MAPKs, therefore inhibiting TLR4 signaling downstream pro-inflammatory factors.

#### 2.2.4 ACT001 Attenuates Neuropathic Pain and Glial Activation

**Figure 6:**
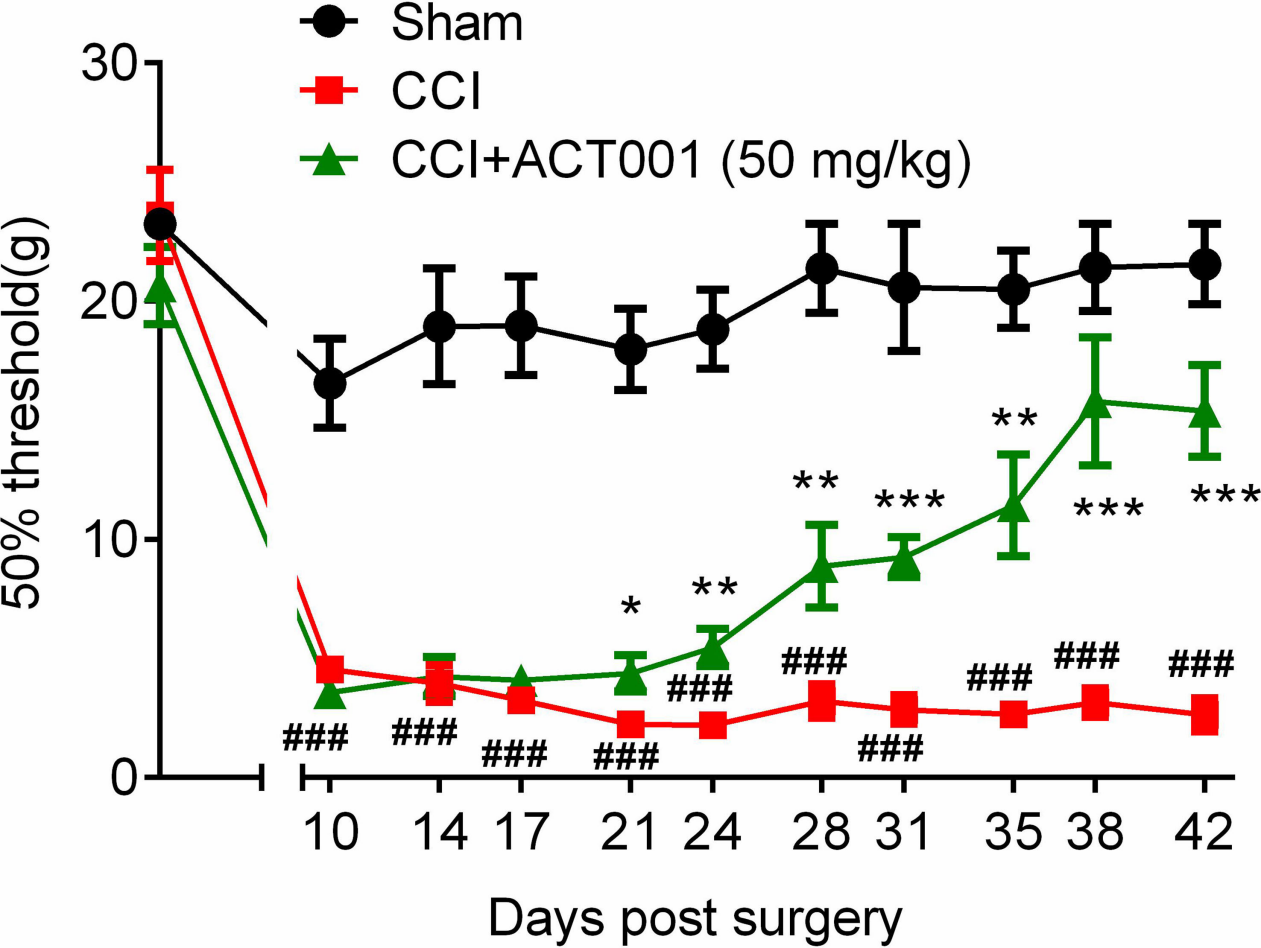
ACT001 attenuates neuropathic pain induced by CCI. All the data represented mean ± SEM. ^###^P< 0.001 *versus* the sham group; *P< 0.05, **P< 0.01, ***P<0.001 versus the CCI group.

**Figure 7:**
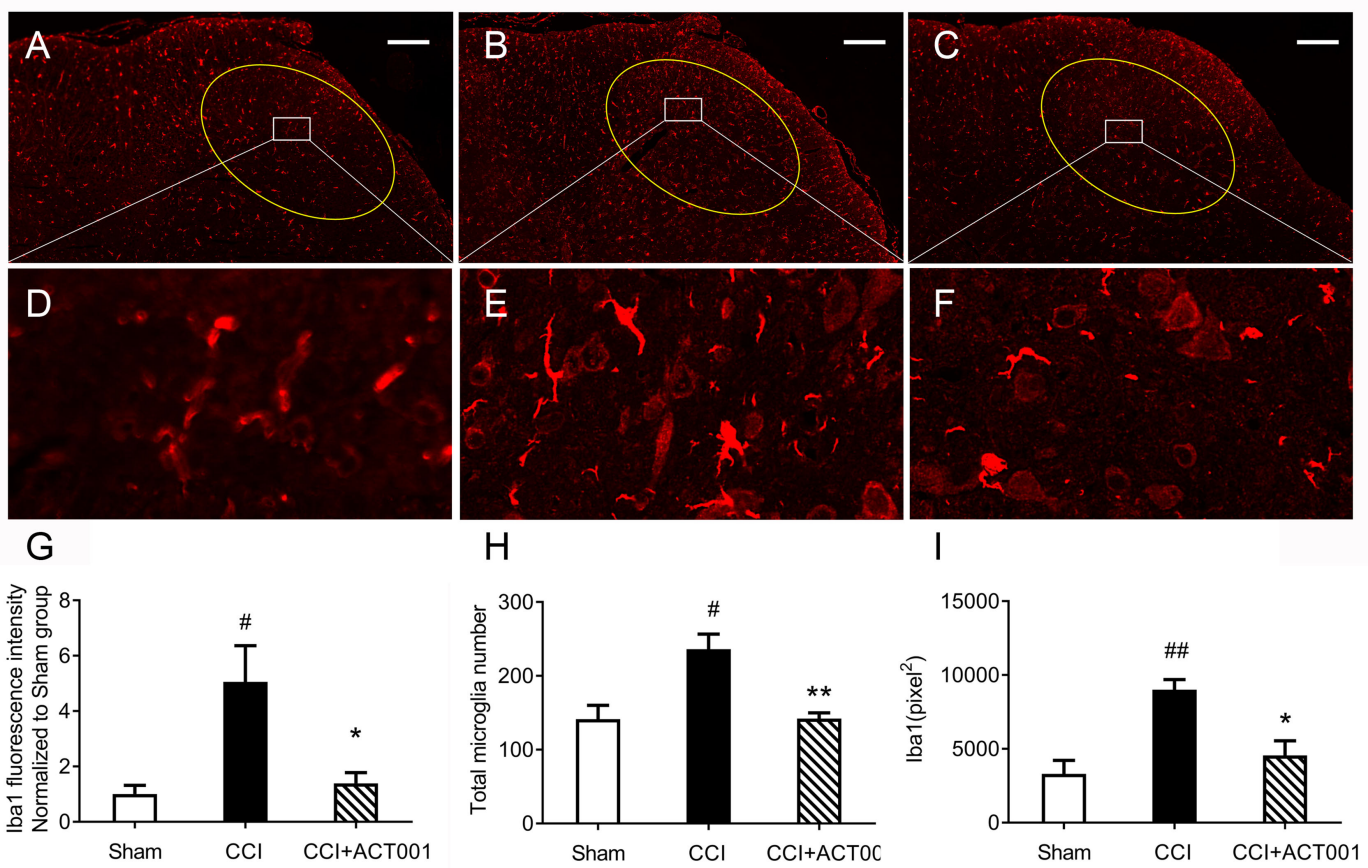
ACT001 inhibits microglial activation markers expression induced by CCI. The lumbar spinal cords (L4-L6) were collected following the final behavioral testing shown in **Figure 6**. These tissues were stained for microglial activation markers. **(A-C)** Representative immunofluorescence Iba1 images for the sham group **(A)**, CCI group **(B)**, and CCI + ACT001 group **(C)**. **(D-F)** Representative images for sham group **(D)**, CCI group **(E),** and CCI + ACT001 group **(F)** with high magnification (×60). **(G-I)** The quantification of Iba1 expression was shown as normalized fluorescence intensity **(G)**, number **(H)**, and size of **(I)** microglia. Scale bar = 200 μm. All the data represented mean ± SEM. ^#^P< 0.05, ^##^P< 0.01 *versus* the sham group; *P< 0.05, **P< 0.01 *versus* the CCI group.

**Figure 8:**
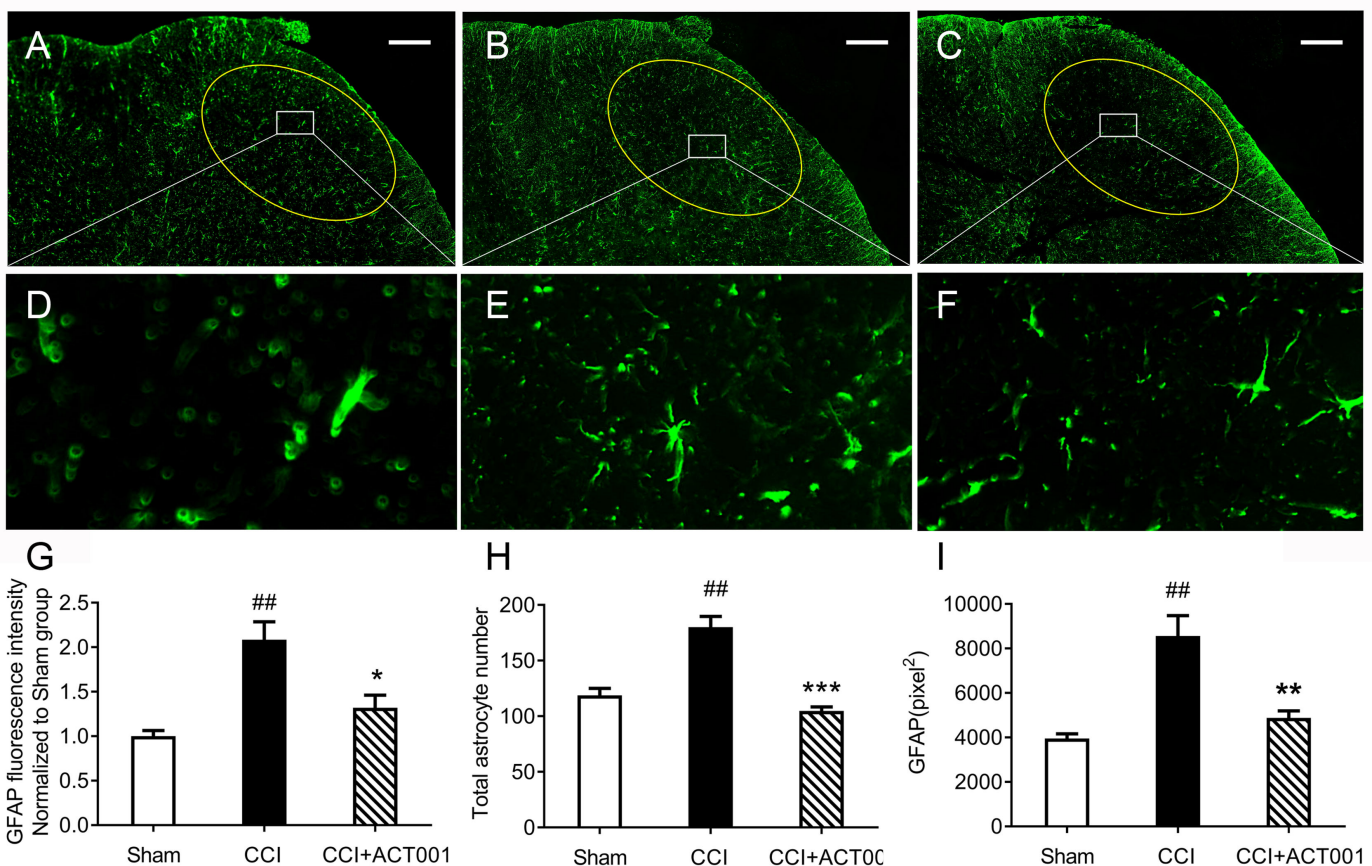
ACT001 inhibits astrocyte activation markers expression induced by CCI. The lumbar spinal cords (L4-L6) were collected following the final behavioral testing shown in **Figure 6**. These tissues were stained for astrocyte activation markers. **(A-C)** Representative immunofluorescence GFAP images for the sham group **(A)**, CCI group **(B),** and CCI + ACT001 group **(C)**. **(D-F)** Representative images for sham group **(D)**, CCI group **(E),** and CCI + ACT001 group **(F)** with high magnification (×60).**(G-I)** The quantification of GFAP expression was shown as normalized fluorescence intensity **(G)**, number **(H)** and size of **(I)** astrocyte. Scale bar = 200 μm. All the data represented mean ± SEM. ^##^P< 0.01 *versus* the sham group; *P< 0.05, **P< 0.01, *** P< 0.001 versus the CCI group.

Chronic constriction injury (CCI)-induced allodynia is associated with up-regulated TLR4 expression in spinal cord and TLR4 antagonism has been shown to attenuate neuropathic pain (41).The mechanical threshold of ipsilateral hind paw of the CCI group decreased significantly compared to the sham group (**Figure 6**). Repeated systemic administration of ACT001 resulted in significant attenuation of allodynia since the 21^st^day after CCI surgery without interrupting weight gain in animals **(** **Figure 6** and **Supplementary Figure 2** **)**. To further analyze whether the attenuation of allodynia by ACT001 was associated with a decrease in the expression of glial activation markers, the lumbar spinal cords (L4-L6) of three group animals were collected following the final behavioral testing. These tissues were stained for microglia and astrocyte activation markers Iba-1 and GFAP, respectively. The expression of Iba-1 and GFAP in the ipsilateral spinal dorsal horn segments in the CCI group significantly increased when compared to the sham group (**Figures 7**, **8**). Moreover, the number and size of glia were also elevated in CCI group compared to sham group. ACT001 significantly inhibited the CCI-induced overexpression of glial markers as well as the Iba1- and GFAP-positive area and the cell number (**Figures 7**, **8**). These results show ACT001 attenuates neuropathic pain and glial activation.

### 2.3 Discussion

ACT001 has been proved to exert anti-tumor functions through a variety of pharmacological activities. Hou*et al* and Tong *et al.* reported that ACT001 inhibited glioblastoma growth by inhibiting AEBP1/PI3K/AKT and signal transducer and activator of transcription 3 (STAT3) signaling pathways (12, 50). Xu et al. found that ACT001 had an anti-growth effect on rhabdomyosarcoma, which was mediated by Bim protein up-regulation and ROS overproduction (51). Ba et al. and Yao et al. discovered that ACT001 repressed hepatocellular carcinoma and osteosarcoma proliferation and triggered cell cycle arrest *via* the intrinsic apoptotic pathway (13, 52). Moreover, ACT001 was found to alleviate NLRP3-mediated neuroinflammation (16). In this study, *in vitro* quenching titrations of MD2 intrinsic fluorescence and STD-NMR demonstrated the direct binding of ACT001 to TLR4 co-receptor MD2. CETSA validated that MD2 is the endogenous target of ACT001 in the cellular context. The RMSF analysis indicated that ACT001 destabilized MD2, which is consistent with CETSA data. In brief, ACT001 was found to bind to MD2 directly and inhibited the formation of TLR4/MD2/MyD88 complex and the TLR4 signaling NF-κB and MAPKs, therefore suppressing neuroinflammation. This study demonstrates that ACT001 is a TLR4 antagonist for the first time, which at least in part accounts for its anti-neuroinflammatory activity. However, it should be acknowledged that most likely ACT001 has other unknown non-MD2 targets, which deserves further investigations.

Neuropathic pain has affected 7%-10% of the general population (53). Despite several therapeutics are available for treating neuropathic pain, they have serious side effects. For example, opioids are less effective in treating neuropathic pain as the negative effects of tolerance and addiction may prevent their long-term use (54). Ion channel blockers such as gabapentin and pregabalin could relieve neuropathic pain, but there are dose limitations concerning efficacy and side effects like dizziness, sedation, and weight gain (55). Ziconotide is an effective analgesic for severe chronic pain refractory to other treatments but only can be delivered intrathecally (55). Therefore, there is an urgent need for the development of therapeutic agent for treating neuropathic pain. Extensive studies have demonstrated that the contribution of activated glia and their pro-inflammatory products to allodynia and that TLR4/MD-2 could be a novel drug target for treating neuropathic pain (7, 8, 56). Consequently, several TLR4 antagonists have been developed (6, 57, 58). However, few of these could cross BBB. Herein, ACT001, which can diffuse through BBB after oral administration (9), was repositioned as a TLR4 antagonist to attenuate allodynia in a preclinical model of neuropathic pain. The systemic administration of ACT001 resulted in antagonism of TLR4-expressing glial cells in the lumbar spinal dorsal horn. The *in vivo* studies provide support for ACT001 as a novel therapeutic drug for chronic pain, which would expand the clinical application of ACT001. It should be acknowledged that the *in vivo* behavioral testing in this study lacked dose-dependent investigation and was performed only in male rats. Further studies should be considered to evaluate the effect of ACT001 with different doses *in vivo* and also evaluate the sex differences of ACT001 as a TLR4 antagonist for treating neuropathic pain.

In summary, this study provides the first evidence that ACT001 binds to MD2, therefore blocking the TLR4 signaling. Furthermore, ACT001 attenuates allodynia induced by CCI and glial activation in dorsal horn of lumbar spinal cord. The results indicate that ACT001 could be a potential therapeutic intervention for chronic neuropathic pain. Our results add that MD2 is one of the important targets of ACT001and can partially explain its interference of innate immune function in CNS diseases.

## Data Availability Statement

The original contributions presented in the study are included in the article/**Supplementary Material**. Further inquiries can be directed to the corresponding authors.

## Ethics Statement

All the animal-handling procedures were approved by the Institutional Animal Care and Use Committee (IACUC) of Changchun Institute of Applied Chemistry, Chinese Academy of Sciences (CIAC2021-0026).

## Author Contributions

XW and YP designed the experiments; SW, SJ, CL, and TZ performed the experiments, acquired and analyzed data; TZ and CL wrote the manuscript; XL and XW edited the manuscript. All authors contributed to the article and approved the submitted version.

## Funding

This work was supported by the National Natural Science Foundation of China (91956121, 21877106); the Chinese Academy of Sciences (CAS) Pioneer Hundred Talents Program; the State Key Laboratory of Natural and Biomimetic Drugs (K202115); Beijing National Laboratory for Molecular Sciences (BNLMS202108).

## Conflict of Interest

The authors declare that the research was conducted in the absence of any commercial or financial relationships that could be construed as a potential conflict of interest.

## Publisher’s Note

All claims expressed in this article are solely those of the authors and do not necessarily represent those of their affiliated organizations, or those of the publisher, the editors and the reviewers. Any product that may be evaluated in this article, or claim that may be made by its manufacturer, is not guaranteed or endorsed by the publisher.
