# Supplementary figures and images for: ACT001 Inhibits TLR4 Signaling by Targeting Co-Receptor MD2 and Attenuates Neuropathic Pain
Source: Front Immunol. 2022 Jun 9;13:873054. doi: 10.3389/fimmu.2022.873054 (PMC9218074; doi:10.3389/fimmu.2022.873054)

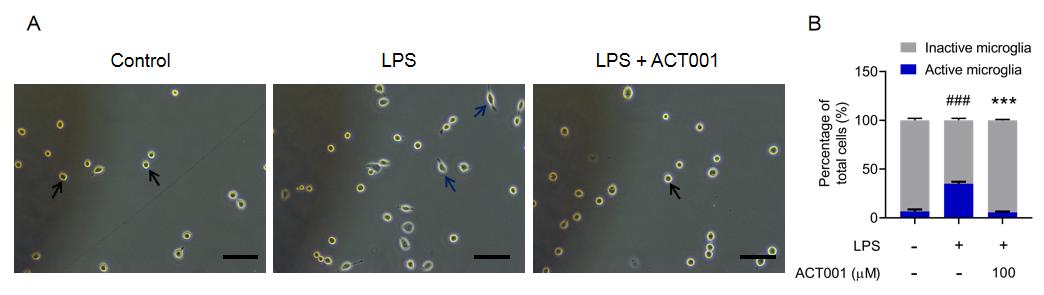

Supplement: Supplementary file 1 [file Image_1.jpeg]

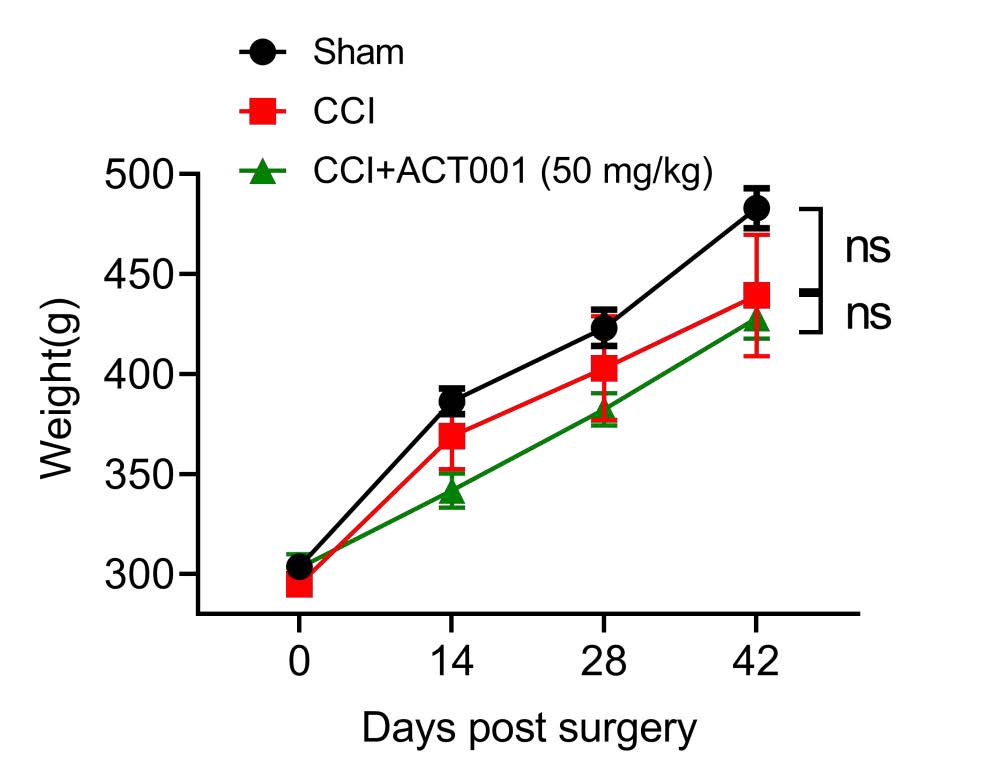

Supplement: Supplementary file 2 [file Image_2.jpeg]
